# Digital tools for at-distance psychiatric support in war time

**DOI:** 10.1192/j.eurpsy.2023.44

**Published:** 2023-07-19

**Authors:** N. O. Maruta

**Affiliations:** boderline disorders department, “Institute of Neurology, Psychiatry and Narcology of the National Academy of Medical Sciences of Ukraine” State Institution, Kharkiv, Ukraine

## Abstract

**Abstract:**

Digital technologies help to improve the work of psychiatric services through the use of modern approaches.

The use of telepsychiatry (TP) during war allows people with psychiatric disorders to receive quality treatment that would otherwise be unavailable.

TP and other digital technologies are an important resource for providing psychiatric care to internally and externally displaced persons affected by war.

As our experience shows, the conditions for effective use of TP are availability of legislative, technical and staff base. The services are implemented according to the protocol, which defines the methods of treatment’s effectiveness evaluation.

The presentation will provide methodological approaches to the use of TP and other digital tools.

**Disclosure of Interest:**

None Declared

